# Green Synthesized Silver Nanoparticles: A Novel Approach for the Enhanced Growth and Yield of Tomato against Early Blight Disease

**DOI:** 10.3390/microorganisms11040886

**Published:** 2023-03-29

**Authors:** Madeeha Ansari, Shakil Ahmed, Asim Abbasi, Najwa A. Hamad, Hayssam M. Ali, Muhammad Tajammal Khan, Inzamam Ul Haq, Qamar uz Zaman

**Affiliations:** 1 Institute of Botany, University of the Punjab, Lahore 54590, Pakistan; 2 Department of Environmental Sciences, Kohsar University Murree, Murree 47150, Pakistan; 3 School of Plant Sciences, University of Arizona, Tucson, AZ 85721, USA; 4 Plant Protection Department, Faculty of Agriculture, Omar Al-Mukhtar University, El-Beida P.O. Box 919, Libya; 5 Department of Botany and Microbiology, College of Science, King Saud University, Riyadh 11451, Saudi Arabia; 6 Division of Science and Technology, Department of Botany, University of Education, Lahore 54770, Pakistan; 7 Department of Entomology, University of Agriculture, Faisalabad 38000, Pakistan; 8 Department of Environmental Sciences, The University of Lahore, Lahore 54590, Pakistan

**Keywords:** silver nanoparticles, plant growth, plant protection, disease management, agricultural sustainability, tomato

## Abstract

Tomato plants are among the most widely cultivated and economically important crops worldwide. Farmers’ major challenge when growing tomatoes is early blight disease caused by *Alternaria solani*, which results in significant yield losses. Silver nanoparticles (AgNPs) have gained popularity recently due to their potential antifungal activity. The present study investigated the potential of green synthesized silver nanoparticles (AgNPs) for enhancing the growth and yield of tomato plants and their resistance against early blight disease. AgNPs were synthesized using leaf extract of the neem tree. Tomato plants treated with AgNPs showed a significant increase in plant height (30%), number of leaves, fresh weight (45%), and dry weight (40%) compared to the control plants. Moreover, the AgNP–treated plants exhibited a significant reduction in disease severity index (DSI) (73%) and disease incidence (DI) (69%) compared to the control plants. Tomato plants treated with 5 and 10 ppm AgNPs reached their maximum levels of photosynthetic pigments and increased the accumulation of certain secondary metabolites compared to the control group. AgNP treatment improved stress tolerance in tomato plants as indicated by higher activities of antioxidant enzymes such as PO (60%), PPO (65%), PAL (65.5%), SOD (65.3%), CAT (53.8%), and APX (73%). These results suggest that using green synthesized AgNPs is a promising approach for enhancing the growth and yield of tomato plants and protecting them against early blight disease. Overall, the findings demonstrate the potential of nanotechnology-based solutions for sustainable agriculture and food security.

## 1. Introduction

Tomatoes (*Solanum lycopersicum* L.), belonging to the family Solanaceae, are among the most important and commonly used crops at the domestic and industrial levels [1,2,3]. The productivity of tomatoes is around 200 million tons globally, economically benefitting the farmers [4]. After potatoes and sweet potatoes, it is the largest vegetable crop in the world [5]. In the food sector, tomatoes are used in a variety of ways. They are used to make soups, salads, pickles, sausages, ketchup, puree, chutney, jam, and many more goods, both for fresh consumption and for processing [6]. The tomato has a strong therapeutic value as a blood cleanser, and it stimulates gastrointestinal secretion. It is a natural stimulant that aids in lowering the amount of toxins present in the blood system [7]. It is popular as it gives food a variety of hues and tastes while also adding vitamin C. Due to its dietary relevance and nutritional worth, it has particular significance [8]. Tomatoes are susceptible to several diseases caused by fungi, bacteria, viruses, and nematodes, even before fruit initiation. One of the most severe diseases affecting tomatoes is early blight, which is brought on by *Alternaria solani* (Sorauer) [9]. A large genus of fungi is called *Alternaria* Nees. Its species, particularly *solani* Sorauer, is the most destructive, causing an early blight disease in tomato and potato plants all over the world [10,11]. *A. solani* colonizes the leaves, stem, and fruits [12] and causes up to 78% crop loss belonging to the family Solanaceae. It is commonly seen in warm and humid growing conditions. Symptoms of early blight include circular, dark-brown spots on leaves that eventually turn yellow and dry up and similar spots on fruit that can lead to rot [13]. In order to maximize tomato production, fertilizers and fungicides are applied in large quantities. The increased use of these pesticides is harmful to humans and other forms of life. The harmful effects of pesticides depend on their toxicity level, contamination level, and exposure duration [14]. The amount of pesticides applied to crops in agricultural fields reached 2.5 million tons annually [15]. Additional methods to combat this deadly disease include the use of resistant cultivars and chemical treatments and the development of systemic acquired resistance in plants. Traditional approaches have been limited by climate change and the rise of diseases with resistance. Therefore, there is a need for tools that are both extremely effective and safe for the environment in order to protect the environment from the damaging effects of pesticides [16].

Nanotechnology has the potential to revolutionize the agricultural industry with novel tools for disease management and rapid disease detection and by improving plant nutrient uptake. It provides innovative tools for delivering agrochemicals safely and without disrupting the ecosystem. It provides carrier systems that can control the release of pesticides at specific locations. It has the potential to reduce the concentration of pesticides in the environment [17]. Because of their mechanical, optical, antibacterial, and antiviral [18] properties as well as their electrical conductivity and thermal conductivity, silver nanoparticles are among the most promising nanomaterials [19,20]. Current projections indicate that agriculture will require considerable gains in crop yields over the next 30 years, which will require the application of AgNPs to improve soil quality [21], pesticide function, and plant development [22]. The size, type, and concentration of nanoparticles all influence how they affect plants [23]. The plant species, its growing conditions, and its sail properties and the bioavailability of AgNPs in the soil also affect the activity of silver nanoparticles. The silver nanoparticle’s impact on edible crop plants should be evaluated before commercializing the product in markets worldwide [24]. Applications of nanomaterials, primarily biological applications, depends on their synthesis methods. Various approaches, such as physical, chemical, and biological, have been developed to synthesize a controlled size and shape and stable nanomaterials [25]. Physical synthesis methods for nanomaterials include physical vapor deposition, ball milling [26], Sol-Gel process [27], laser ablation [28], and electron beam evaporation [29], but these methods require costly equipment and infrastructure. There is poor reproducibility due to the sensitivity of physical methods to process parameters, the limited control over particle size and composition, difficulties in scaling up the synthesis process for commercial production, and the generation of hazardous waste during the process, which may require proper disposal [30]. Chemical synthesis methods include hydrothermal synthesis [31], coprecipitation [32], chemical reduction [33], microemulsion [34], and solvothermal synthesis [35]. However, these processes use synthetic chemicals of high cost and generate hazardous chemicals and by-products during the synthesis process [36].

In comparison, biological methods employ microorganisms [37], such as cell cultures of bacteria, fungi, and plants [38]. Plants are preferable to other biological sources for nanoparticle synthesis due to the simple and one-step procedure that avoids the time-consuming process of maintaining cell culture and a non-aseptic environment [39]. Plants are widely available and can be sourced locally, reducing the need for imported materials. They are easy to handle, are a rich source of many metabolites, and include pharmacological ingredients that work as reducing and capping agents in the creation of nanoparticles, preventing the development of undesirable by-products and enhancing nanoparticle stability [40]. Using this procedure, one may synthesize nanoparticles with unique properties such as improved stability, biocompatibility, and antibacterial activity that are both economical and environmentally benign [41,42]. Nanoparticles synthesized using plants have many applications, including in biomedical, environmental, and energy-related fields [43]. Traditional chemical synthesis methods often require hazardous chemicals, but plant-mediated synthesis eliminates this need, making the process safer and more sustainable [44].

Common plants used in the synthesis of nanoparticles include *Azadirachta indica* [45], *Ocimum basilicum* [46], *Moringa oleifera* [47], and *Citrus limon* [48]. This study aimed to develop a green method for synthesizing silver nanoparticles and investigated their efficacy as a nano-fungicide against early blight in tomato plants. The impacts of silver nanoparticles on the growth rates, biomass output, yield, and certain biochemical traits of the plants were assessed during the interaction between the plants and nanoparticles.

## 2. Materials and Methods

### 2.1. Materials

Leaves of the neem plant were collected to prepare the plant extract for the synthesis of silver nanoparticles (AgNPs). Seeds of two tomato varieties named nadar and naqeeb were brought from the vegetable research institute of the Ayyub Agricultural Research Institute (AARI) in Faisalabad. The required chemicals were purchased through Science Traders from Sigma-Aldrich, St. Louis, MO, USA.

### 2.2. Synthesis of Silver Nanoparticles

The Ahmed et al. [49] modified procedure was used to prepare the plant extract. The leaves of the neem plant were washed, dried at room temperature under shade, and crushed with an electric grinder to make a powder material. The powder material in an amount of 10 g was added to 100 mL of distilled water, brought to a boil for 30 min, allowed to cool, and then filtered through Whatman’s No. 1. Then, 20 mL of the leaf extract was mixed with 10 mL of 1 mM silver nitrate in a vial and incubated for 3 h at 70 °C. The color of the reaction mixture changed, and the resultant solution was examined using UV-vis spectroscopy with a wavelength range of 300–800 nm (model BMS: 2800). The reaction mixture was taken in Eppendorf’s tube and centrifuged at 10,000 rpm for 10 min. Prior to obtaining pure AgNPs, the centrifugation cycle was repeated several times. Silver nanoparticles were kept in storage at 4 °C for further use.

### 2.3. Source of Alternaria solani

The isolate of *Alternaria solani* L. was isolated from the infected leaf of a tomato plant with the accession number OP984330.

### 2.4. In Vitro Antifungal Studies of Silver Nanoparticles

Commercially available potato dextrose agar (PDA) media of Sigma-Aldrich was used to prepare Petri plates for the antifungal activity of AgNPs. To inhibit the bacterial growth, a 2% solution of streptomycin was applied to the medium. A standard solution of pure AgNPs was used to make concentrations of 5 ppm, 10 ppm, 15 ppm, 20 ppm, 25 ppm, and 50 ppm. The PDA media and 1 mL solution of different concentrations of AgNPs were poured into respective Petri plates and incubated at 25 ± 1 °C, 75 ± 5% R. H for 48 h. The plate without AgNP solution was taken as a control. An agar plug of equal size was placed in the center of each Petri plate, using a sterile cork borer from the pure culture of *A. solani* incubated for 14 days at ±27 °C in an incubator (model WIG-32, WiseCube Co. Ltd., Seoul, Korea). The experiment was performed in triplicate, and the plates were labeled. All the work was performed in a laminar airflow cabinet under a sterilized environment to avoid contamination. The radial growth of the colony was measured, and the rate of inhibition was calculated using the following formula:

(1) $$\mathrm{Rate}\;\mathrm{of}\;\mathrm{Inhibition}\;\left(\%\right)=\frac{R-r}{R}\times 100$$

where “R” denotes the expansion of the fungus’ mycelium on control plates, and “r” denotes the expansion of the fungus’ mycelium on AgNP-treated plates [50].

### 2.5. In Vivo Antifungal Studies of Silver Nanoparticles

#### 2.5.1. Experimental Design

A pot experiment was performed to determine the potential of AgNPs against the early blight disease of tomato caused by *A. solani*. Using a randomized complete block design (RCBD) with three replicates, the experiment was conducted on the highly susceptible varieties nadar and naqeeb. Loamy soil and farmyard manure were mixed to prepare the pots. To prevent soil water loss and excessive drainage, pebbles were used to partially plug the pores at the bottom of the pots. The pots were filled with 7 kg/pot soil and were arranged according to varieties, respective treatments, and replicate numbers, and they were provided similar environmental conditions (25 ± 1 °C, 75 ± 5% R. H and 12 h photoperiod). Already prepared seedlings of tomato varieties were transplanted in prepared pots, and one plant per pot was kept. Standard agricultural practices were used to irrigate and maintain the plants. Weeds were removed manually during the experimentation. The pots were regularly examined throughout the season.

#### 2.5.2. Inoculation Preparation and Application of *A. solani*

The conidial suspension was prepared from the 10-day-old culture of *A. solani*. To collect the conidia, the plates were flooded with ddH_2_O (Fisher Scientific, 0.01% Tween 20 surfactant, Waltham, MA, USA). The prepared conidial suspension at a rate of 10^3^ mL^−1^ in water was sprayed on leaves until runoff [51].

#### 2.5.3. Application of Silver Nanoparticles

Six different concentrations of AgNPs, i.e., 5, 10, 15, 20, 25, and 50 ppm, were sprayed until runoff on leaves of tomato plants at intervals of 15 days till harvest. The first treatment was given 35 (vegetative stage 1 according to BBCH scale) days after transplantation (DAT). Positive controls were provided with the standard dose (6 ppm) of commercially available fungicide (cabrio top). The active ingredients in the cabrio top are pyraclostrobin and metriram, which work together and provide both preventative and curative protection against a range of fungal pathogens. Tap water was given to plants taken as the negative control.

#### 2.5.4. Measurement of Disease Parameters of Tomato Plant

The disease parameters, i.e., lesion size, number of lesions/plant, and disease incidence, were recorded at 55 DAT (vegetative stage 19, according to BBCH scale) and 85 DAT (inflorescence emergence stage 5, according to BBCH scale).

#### 2.5.5. Growth and Yield Analysis of Tomato Plant

In order to evaluate the effects of NPs on plant development and biomass yield at the vegetative, reproductive, and mature phases, plants protected against disease attack by AgNPs were also examined. The plants at vegetative stage 19 and inflorescence emergence stage 5 were taken out of the pots, intact with their roots, and were brought in bags to the lab to measure the morphological characteristics (shoot length, root length, number of leaves, and leaf area) and fresh biomass. Yield characteristics, including fruit fresh weight and size, number of fruits/plant, and fruit yield, were measured at fruit ripening stage 88 according to the BBCH scale. To assess how AgNPs affected the growth of dry biomass, the plants were dried. The plants were placed in paper bags and dried for 3 days in a Wiseven oven (Model WOF-105, Korea) at a temperature of 70 °C, and the weights were measured with a digital weighing balance (Cubis_II_analytical balance, Sartorius, Göttingen, Germany).

#### 2.5.6. Determination of Photosynthetic Pigments of Tomato Plant

Healthy leaves were taken from potted plants at 55 and 85 DAT. The measurements of the photosynthetic pigments, including chlorophyll a and b, were measured in accordance with the instructions provided by Davies [52] and Arnon [53]. The Kirk and Allen [54] approach was used to determine the sample’s carotene content (1965).

#### 2.5.7. Quantification of Alkaloids, Flavonoids, Total Soluble Sugar, and Total Soluble Protein of Tomato Plant

Healthy leaves were taken from potted plants at the vegetative and reproductive stage DAT. Three samples were taken for each treatment.

The alkaloids were quantified using the method of Ulubelen et al. [55] by thin layer chromatography (TLC). Alkaloids were extracted from 2 g samples of the dried plant organs with n-hexane (3 × 20 mL) followed by MeOH (3 × 20 mL). The extraction was conducted at room temperature, and each cycle with hexane or MeOH lasted 24 h. The extract was evaporated in a rotary film evaporator (RFE). As the last step, each tube was rinsed with 15 mL of distilled water, which was added to the flask. The non-alkaloids in the mixture were removed with CH_2_Cl_2_ (3 × 1/3 of the total volume). The acidic aqueous solution was made alkaline (pH 8 to 10) by adding 10% NaOH, and the contents were extracted with CH_2_C1_2_ to obtain the alkaloids [55]. At this stage, the aqueous phase was discarded, and the organic phase was dried in RFE. The residue was dissolved in 1 mL CH_2_C1_2_ and stored until further use.

With a few adjustments, the approach of Krizek et al. [56] was used to determine the total flavonoid content in the leaves. Briefly, 1 g of leaves was ground with 3 mL of 1% acetic acid-ethanol (1:99, *v*:*v*), followed by centrifugation at 12,000× *g* for 15 min. The supernatant was transferred into a polypropylene tube and incubated in a water bath at 80 °C for 10 min. The spectrophotometer was used to measure the absorbance at 270, 300, and 330 nm. Total flavonoid content was calculated using the extinction coefficient of 0.033 mM/cm.

Using the Nelson [57] procedure, the total amount of soluble sugar was calculated (1944). Fresh leaf in the amount of 0.5 g was taken in 10 mL of alcohol for 60 min at 60 °C and filtered to estimate the total soluble sugar. The remaining plant residues were again mixed in alcohol, and the volume was made to 25 mL. An amount of 1 mL of extract was mixed with 1 mL of 5% phenol and 5 mL sulfuric acid. Using 80% ethanol as a blank, the absorbance at 485 nm was measured using a spectrophotometer.

The Lowry [58] technique was used to determine the total soluble protein content. By mixing phosphate buffer (0.1 M, pH-7, 1:4 (*w*/*v*)) in a pre-chilled pestle and mortar, frozen leaves were crushed. For 1 g of plant leaves, 4 ml of phosphate buffer was added. Then, at 10,000 rpm at 4 °C, the sample was centrifuged for 10 min. In 0.4 mL of supernatant, 0.2 mL of Folin’s mixture was poured, and at room temperature, the test tube was placed for 15 min. Then, for the development of color, 0.2 mL of Folin’s Phenol reagent was added, mixed, and placed at room temperature for 45 min. A double-beam spectrophotometer was used to measure the absorbance at 750 nm.

#### 2.5.8. Assessment of Proline and Phenolics Activity of Tomato Plant

The proline concentration was assessed spectrophotometrically at 520 nm after the combination was extracted with toluene, in accordance with the Bates [59] ninhydrin technique. In 3 mL of 3% sulfosalicylic acid, fresh leaf samples (300 mg) were homogenized. Filtrate was poured in test tubes and placed in a water bath at 100 °C, and then, it was reacted with 1 mL each of acid ninhydrin and glacial acetic acid for 1 h. By using L-pro as a standard, the absorbance was measured spectrophotometrically at 520 nm after the extraction of the mixture with toluene.

Using the method described by Lim et al. [60], the Folin–Ciocalteu reagent was used to assess the total phenols in plant extracts after treatment. By using methanol in the amounts of 0.01, 0.02, 0.03, 0.04, and 0.05 mg/g, the concentration of gallic acid was prepared. In methanol in the amount of 0.1 ml, the plant extract was also prepared. In all test tubes of 0.5 mL samples, 2 mL of 7.5% sodium carbonate and 2.5 mL of Folin reagent were added. By using parafilm, test tubes were sealed and placed at room temperature for 30 min. The absorbance was measured at 760 nm using a spectrophotometer. The Folin–Ciocalteu reagent created a blue color after the reaction due to its sensitivity to the reducing compounds, including polyphenols.

#### 2.5.9. Estimation of Antioxidative Activities of Tomato Plant

The superoxide dismutase (SOD) activity was evaluated using the method developed by Sairam et al. [61]. Using the unit/mg protein/30 min method, the SOD activity was determined. For that purpose, 2 g of leaf sample was mixed with a buffer solution (0.05 M phosphate buffer), ascorbic acid, and polyvinylpyrrolidone with a pH of 7.0. The mixture was then centrifuged at 12,000 rpm for ½ hour at 4 °C. The reaction mixture was composed of 100 μL of Na_2_CO_3_, 200 μL if methionine, 100 μL of NBT, 100 μL of EDTA, 1.5 mL of potassium buffer (pH 7.5), 1.0 mL of d/w, and 150 μL of enzyme.

To assess the peroxidase (PO) activity of treated plants, the Fu and Huang [62] methodology was employed. A total of 470 nm/g fresh weight/minute was used to express the activity of PO. Leaf samples (0.2 g), completely washed, were kept frozen using liquid nitrogen and were crushed with mortar and pestle in 5 mL of 100 mM phosphate buffer solution until homogenized. The solution was then centrifuged for 25 min at 12,000 rpm. The obtained supernatant was taken in a beaker and allowed to react with 20 μL of 40 mM hydrogen per oxide. The oxidation of guaiacol was noted at 470 nm min^−1^ with the help of a spectrophotometer.

The Mayer et al. [63] approach was used to gauge the activity of polyphenol oxidase (PPO). The enzyme activity was measured using the substrate catechol. To make the reaction mixture, 1.5 mL of 10 mM sodium phosphate buffer (pH 6.0) was added to 150 mL of 0.1 M catechol solution. Following that, 200 mL of the enzyme mixture was added to the tube and incubated for 1 h at room temperature. PPO activity was calculated to be 495 nm/min/mg protein based on the observed absorbance at 495 nm.

The phenylalanine ammonia-lyase (PAL) activity was evaluated using the procedure developed by Burrell and Rees [64]. In micrograms of trans-cinnamic acid per hour per milligram of protein, the enzyme activity was calculated. L phenylalanine (250 mL and 0.03 M) was added in 2.5 mL of sodium borate (Na_2_H_20_B_4_O_17_) buffer and 200 mL of the reaction mixture and maintained at a pH of 8.8. For 1 h, this reaction mixture was placed in a 37 °C water bath. After incubation, 1 M of trichloroacetic acid (C_2_HCl_3_O_2_) solution was added, and the absorption rate was determined at D290.

Using a modified version of the Nakano and Asada [65] approach, the ascorbate peroxidase (APX) activity of the relevant material was measured. First of all, 2 g of leaf sample was mixed with a buffer solution (0.05 M phosphate buffer), ascorbic acid, and polyvinylpyrrolidone with a pH of 7.0. The mixture was then centrifuged at 12,000 rpm for 30 min at 4 °C. For the assay of enzyme activity, the rate of the hydrogen peroxide-dependent oxidation of ascorbic acid was determined in a reaction mixture that contained 0.25 mL of 0.05 M phosphate buffer with a pH of 7.0, 0.25 mL of 0.0001 M ethylenediamine tetra-acetic acid (EDTA), 0.25 mL of 0.05 M ascorbate, and 150 μL of supernatant. The reaction was initiated by the addition of 0.25 mL of 0.0001 M H_2_O_2_, and the oxidation rate of ascorbic acid was estimated by following the decrease in absorbance at 290 nm for 1 min. Using an attenuation value of 2.8 mM^−1^ cm^−1^, the APX activity was measured as mol ascorbate oxi./mg protein/2 min.

### 2.6. Data Analysis

The data collected during lab and field work were evaluated by one-way analysis of variance (ANOVA) (ANOVA). Duncan’s multiple range test (DMRT) at the 0.05 percent level of significance was used to separate the treatment means. All statistical analyses were carried out using Statistix 8.1 package (Analytical Software, Tallahassee, FL, USA).

## 3. Results

### 3.1. In Vitro Antifungal Studies of Silver Nanoparticles

**Figure 1:**
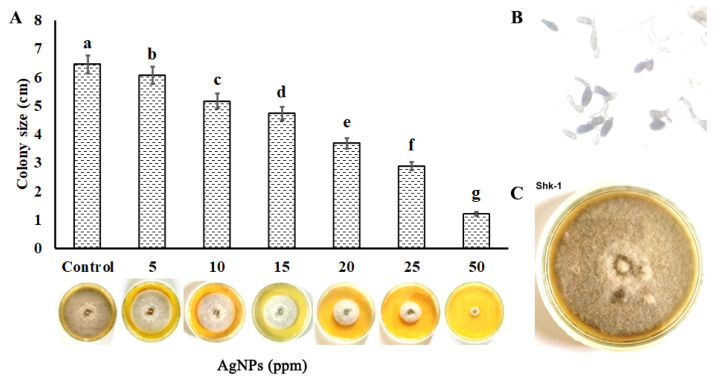
(**A**) The antifungal activity of green synthesized silver nanoparticles against different isolates of *A. solani*, (**B**) Conidia of *A. solani*, and (**C**) Colony of *A. solani*. Standard errors are represented by vertical bars, and letters represent a variation in antifungal activity by treatments using Duncan’s multiple range test.

The radial growth of the tested pathogen was used to assess the antifungal activity of green synthesized silver nanoparticles under in vitro conditions. The results determined the potential of green synthesized silver nanoparticles prepared from the neem plant leaf extract as a nano-fungicide against potential fungal pathogens. It was observed that various concentrations of silver nanoparticles, i.e., 5, 10, 15, 20, 25, and 50 ppm, significantly inhibited the fungal culture growth compared to the control. The inhibition rate increased from 20% to 81% with the increase of the concentration of NPs, as given in Figure 1. The maximum inhibition rate was observed at 50 ppm of AgNPs with a 1.22 ± 0.033 cm zone.

### 3.2. In Vivo Antifungal Studies of Silver Nanoparticles

**Figure 2:**
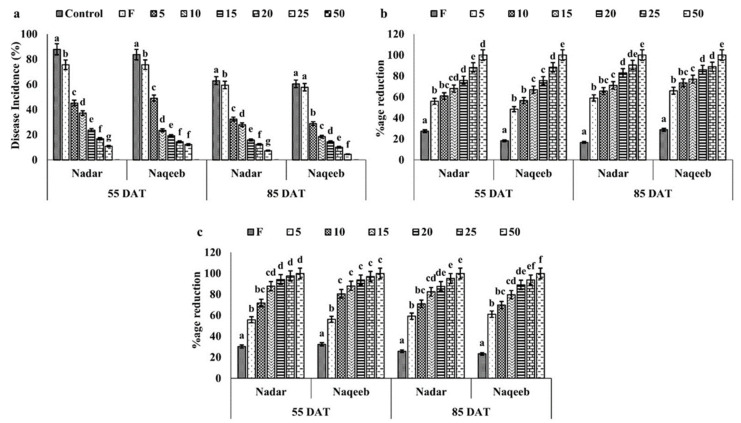
(**a**) The disease suppression efficacy of green synthesized silver nanoparticles against the early blight of tomato plants at vegetative and reproductive stages during the pot experiment: (**a**) disease incidence (%); (**b**) number of lesions/plant (%); and (**c**) lesion size (%). Bars with different lower-case letters are significantly different at *p ≤* 0.05 (Duncan’s multiple range test). Vertical bars indicate standard error (SE) values.

The in vivo antifungal activity of green synthesized silver nanoparticles against *A. solani* was determined in pots under greenhouse conditions. Results demonstrated that green synthesized silver nanoparticles controlled the growth of *A. solani* and played an active role in disease reduction. A 62.92% disease incidence was noted in control plants of the nadar variety after 55 DAT, while 60.46% was in plants of the naqeeb variety. It increased with time and rose to 88% and 83.68% at 85 DAT in plants of both varieties, respectively. The plants sprayed with the standard dose of fungicide reduced the disease incidence by 9% compared to the control and showed up to a 75% disease incidence. The lower concentration of silver nanoparticles, i.e., 5 ppm, reduced the pathogen growth, and the plants showed a 45–49% disease incidence in both varieties at 55 DAT. Further treatments of 5 ppm AgNPs reduced the disease incidence by more than 50%. An increase in the concentration of AgNPs (10–20) inhibited the disease incidence by 40–75% and 60–90% at 55 DAT and 85 DAT, respectively, in both varieties (Figure 2a). The lesion size and their number per plant were also recorded to determine the effect of AgNPs on pathogen growth on the leaves of both varieties of tomato plants. The number of lesions/plant reduced by 50–95% in plants treated with AgNPs compared to the control (Figure 2c), and their size was reduced from 13.96 mm^2^ to 3.71 mm^2^ with 5–25 ppm of AgNPs at 55 DAT and up to 2.95 mm^2^ at 85 DAT (Figure 2b). No symptoms were recorded on the leaves of plants treated with 50 ppm silver nanoparticles. The plants of the naqeeb variety showed a more significant effect of AgNPs than nadar.

### 3.3. Effect of Silver Nanoparticles on Growth Traits of Tomato Plant

The present study also looked at how silver nanoparticles affected tomato plant development characteristics such shoot length, root length, leaf area, and number of leaves measured at 55 and 85 DAT. When compared to the control, the results revealed that tomato plants of both types grew much better when exposed to 10–20 ppm AgNPs (Table 1).

In 30 days under greenhouse circumstances, the shoot and root length of the control plants of the nadar variety increased from 31.58 cm and 18.67 cm to 38.37 cm and 26.57 cm, whereas they increased from 30.96 cm and 16.83 cm to 44.80 cm and 27.77 cm in the case of the naqeeb variety. Plants of both varieties treated with fungicide had shoot and root lengths that were not significantly different from the control. In comparison to the control, the lower concentrations of silver nanoparticles 5 to 20 ppm increased the shoot and root length by 20–80% in both varieties at both stages. Plants of the nadar variety treated with 10 ppm silver nanoparticles at 55 DAT had maximum shoot and root lengths of 52.81 cm and 29.30 cm, respectively, whereas plants of the naqeeb variety had maximum shoot and root lengths of 53.85 cm and 29.30 cm, respectively. Nevertheless, plants treated with 5 ppm silver nanoparticles at 85 DAT showed the longest shoot and root lengths. When AgNP concentrations were increased further to 25 and 50 ppm, the shoot and root lengths were 20% shorter than they were in the control.

As silver nanoparticles were sprayed on the leaves, the quantity of leaves on each plant, along with their size, was counted. With a leaf area of 27.87 cm^2^ for the nadar variety and 29.67 cm^2^ for the naqeeb variety, respectively, the number of leaves in control plants rose by 40% from 55 DAT to 85 DAT. The number of leaves increased by 70% in plants treated with 10 ppm at 55 DAT and 5 ppm at 85 DAT compared to the control in both varieties and reduced up to 15% at higher concentrations, suggesting the lower concentrations and less exposure of silver nanoparticles were more effective in plant growth improvement. The maximum leaf area of 46.10 cm^2^ was recorded in plants of the naqeeb variety with 5 ppm AgNPs at 85 DAT.

### 3.4. Effect of Silver Nanoparticles on Biomass Production of Tomato Plant

The application of green synthesized silver nanoparticles also affected the fresh and dry biomass of the tomato plant. The shoot fresh and dry biomass was 28.71 g and 8.36 g in control plants of the nadar variety at 55 DAT, which increased to 39.63 and 8.24 g at 85 DAT. The silver nanoparticles increased the shoot fresh and dry biomass by 75% with 5 and 10 ppm concentrations at 55 and 85 DAT in both varieties of tomato plants. The maximum fresh and dry biomass of roots at 55 DAT was recorded to be 14.26 g and 5.24 g in plants of the nadar variety treated with 10 ppm silver nanoparticles, which increased up to 21.77 g and 5.47 g, respectively. A similar pattern was followed by plants of the naqeeb variety, as shown in Table 2. The higher concentration of silver nanoparticles (25 and 50 ppm) reduced the fresh and dry biomass in plants of both varieties compared to the control.

### 3.5. Effect of Silver Nanoparticles on Photosynthetic Pigments of Tomato Plant

Green synthesized silver nanoparticles also affected the photosynthetic pigments, including total chlorophyll content, chlorophyll a and b, and carotenoids (Table 3). The results demonstrated that lower concentrations of silver nanoparticles (5–10 ppm) improved the photosynthetic pigments of both varieties by more than 50%. The higher concentrations of AgNPs, such as 25 and 50 ppm, reduced chlorophyll and carotenoids, resulting in less food production and plant growth.

### 3.6. Effect of Silver Nanoparticles on Alkaloids, Flavonoids, Total Soluble Sugar, and Total Soluble Protein of Tomato Plant

**Figure 3:**
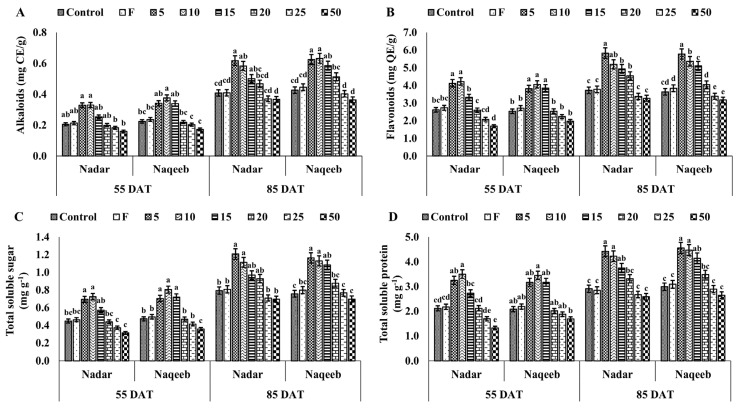
The effect of green synthesized silver nanoparticles on the physiological parameters of tomato plants: (**A**) alkaloids, (**B**) flavonoids, (**C**) total soluble sugar, and (**D**) total soluble protein. Bars with different lower-case letters are significantly different at *p ≤* 0.05 (Duncan’s multiple range test). Vertical bars indicate standard error (SE) values.

Silver nanoparticles improved the growth of plants and affected the production of various essential biochemicals such as alkaloids, total flavonoids, total soluble sugar, and total soluble protein (Figure 3).

The alkaloid production increased by over 50% with 5 and 10 ppm of silver nanoparticles in both varieties at 55 DAT. The 5 ppm silver nanoparticles increased the amount of alkaloids from 0.33 mg CE/g to 0.583 mg CE/g in plants of the nadar variety and 0.34 mg CE/g to 0.625 mg CE/g within 30 days with 5 ppm silver nanoparticles. The plants of the naqeeb variety produced more alkaloids and were healthier than those of the nadar variety. The minimum production of alkaloids was observed with a 50 ppm concentration of silver nanoparticles in both varieties.

The total flavonoid content also increased significantly when plants of both varieties were exposed to lower concentrations of silver nanoparticles than control plants. The total flavonoid content of the nadar and naqeeb plant control group at 55 DAT was 2.62 mg QE/g and 2.54 mg QE/g, which increased to 3.721 mg QE/g and 3.636 mg QE/g at 85 DAT, respectively. The maximum amount of alkaloids of 4.23 mg QE/g and 4.06 mg QE/g was produced at 55 DAT in nadar and naqeeb variety leaves using 10 ppm concentrations. With further treatments of AgNPs, the maximum amount of alkaloids of 5.833 mg QE/g and 5.778 mg QE/g was produced using 5 ppm in leaves of nadar and naqeeb varieties.

Figure 3c shows the significant change in total soluble sugar production in both varieties of tomato plants. The maximum production of total soluble sugar was 0.724 mg g^−1^ and 0.805 mg g^−1^ in plants of the nadar and naqeeb variety, respectively, using 10 ppm AgNPs, which was more than 60% compared to the control at 55 DAT. The production of total soluble sugar was improved with further treatments of AgNPs in both varieties at 85 DAT. The higher concentrations of AgNPs reduced its production compared to the control.

A similar trend was observed in the accumulation of protein content in both varieties of tomato plants. The lower concentrations of AgNPs (5–15 ppm) produced more protein content in both varieties than in the control plants. On the other hand, higher concentrations reduced the protein content at 55 and 85 DAT, as shown in Figure 3d.

### 3.7. Effect of Silver Nanoparticles on Total Phenolic Content and Stress Enzymes of Tomato Plant

**Figure 4:**
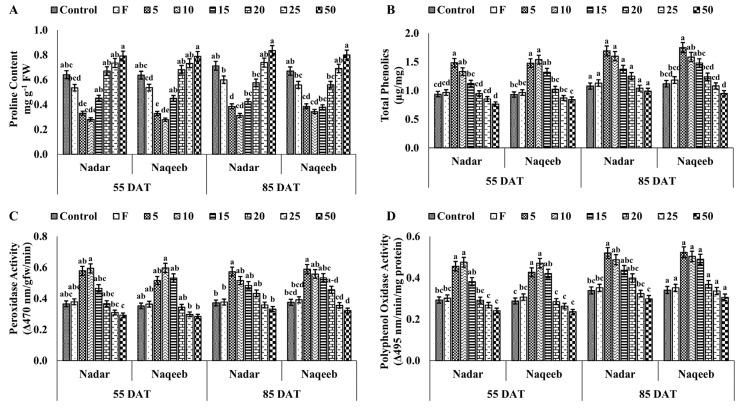
The effect of green synthesized silver nanoparticles on disease-related compounds of tomato plants: (**A**) proline content, (**B**) total phenolics, (**C**) peroxidase activity (PO), and (**D**) polyphenol oxidase (PPO) activity. Bars with different lower-case letters are significantly different at *p ≤* 0.05 (Duncan’s multiple range test). Vertical bars indicate standard error (SE) values.

Silver nanoparticles produced more phenolic content, PO, PPO, PAL, CAT, SOD, and APX to reduce the pathogenic stress in plants. The higher concentrations of AgNPs caused more stress in plants than in the control, producing a higher amount of proline. A lower amount of proline was detected in plants treated with lower concentrations (5–10 ppm), indicating stress reduction, as shown in Figure 4b. The maximum phenolic contents of 1.483 µg mg^−1^, 1.479 µg mg^−1^, 1.691 µg mg^−1,^ and 1.750 µg mg^−1^ were quantified in plants of both varieties treated with 5 ppm AgNPs at 55 and 85 DAT, respectively. The PO activity was also improved in both varieties of tomato with the exposure to lower concentrations of AgNPs compared to the control. More PO activity was observed in plants of the nadar and naqeeb varieties at 55 DAT than 85 DAT, suggesting that the exposure to AgNPs inhibits the pathogen attack, resulting in the production of less reactive oxygen species (ROS), lowering the PO activity. A similar trend was observed in PPO activity, as shown in Figure 4d. The PPO activity ranged from 0.241 to 0.475 in plants of the nadar variety, from 0.236 to 0471 in plants of the naqeeb variety at 55 DAT, from 0.299 to 0.520, and from 0.306 to 0.523 in plants of nadar and naqeeb varieties at 85 DAT, respectively.

**Figure 5:**
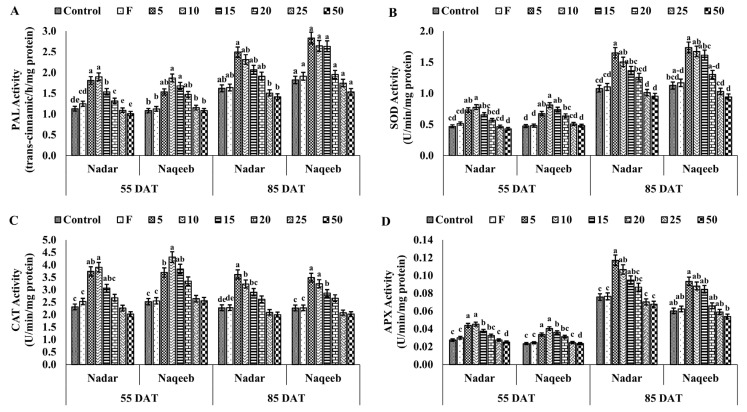
The effect of green synthesized silver nanoparticles on disease-related compounds of tomato plants: (**A**) phenylalanine ammonia lyase (PAL) activity, (**B**) superoxide dismutase (SOD) activity, (**C**) catalase (CAT) activity, and (**D**) ascorbate peroxidase (APX) activity. Bars with different lower-case letters are significantly different at *p ≤* 0.05 (Duncan’s multiple range test). Vertical bars indicate standard error (SE) values.

The highest PAL activity was assessed in tomato plants with lower concentrations of AgNPs. The lower concentrations of AgNPs (5–20 ppm) exhibited more PAL activity of 16.82–67.93% and 35.76–72.92% in nadar and naqeeb variety plants, respectively, at 55 DAT compared to the control. Figure 5a suggests that further treatments of AgNPs improved the PAL activity after 30 days. The production of superoxide dismutase (SOD) was induced by applying silver nanoparticles in a lower concentration. The activity of SOD was increased up to 65.31% and 71% with 10 ppm AgNPs at the first stage in plants of nadar and naqeeb varieties, respectively, compared to the control. A further increase in concentrations started in the reduction of SOD activity, indicating the severe stress conditions in plants.

Catalase (CAT) is another important antioxidant enzyme that reduces plant stress-induced toxicity. In contrast to the control and fungicide, the activity of CAT was markedly increased in plants treated with AgNPs. In comparison to the control, plants exposed to lower concentrations of silver nanoparticles (5–15) had more activity. Nevertheless, at both stages and in both kinds, activity began to decline with an increase in NP concentration. The maximum activity of 3.903 and 4.313 was recorded with 10 ppm AgNPs at 55 DAT and 3.615 and 3.485 at 85 DAT in nadar and naqeeb plants, respectively. The control plants showed activity within the range of 2.266–2.521, and the lowest activity was demonstrated with 50 ppm AgNPs. A similar trend was observed with APX activity in plants of both varieties. The activity was increased by more than 50% from 0.027 to 0.045 and 0.040 with 10 ppm AgNPs in nadar and naqeeb variety plants at the first stage, respectively. The higher concentrations of silver nanoparticles reduced the activity, creating more stress in plants at both stages. The above-mentioned results indicate that AgNPs induce more resistance in plants against *A. solani* than the standard dose of used fungicide.

### 3.8. Effect of Silver Nanoparticles on Yield Attributes of Tomato Plant

**Figure 6:**
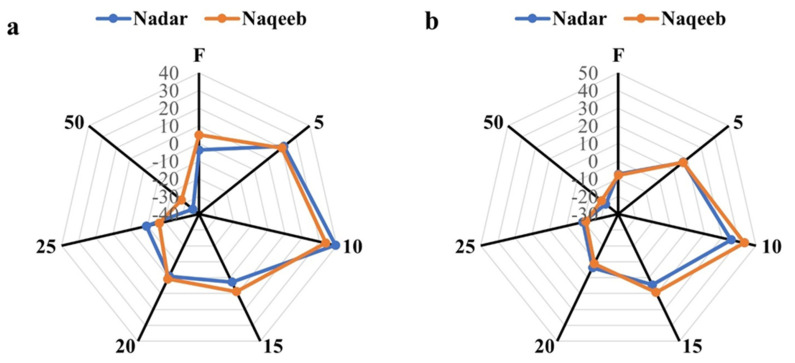
The effect of green synthesized silver nanoparticles on yield parameters of tomato plants: (**a**) fruit size (%) and (**b**) tomato yield (%).

**Figure 7:**
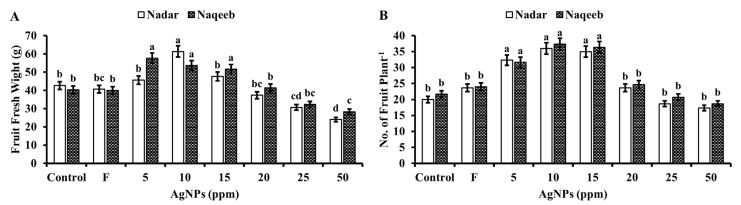
The effect of green synthesized silver nanoparticles on yield parameters of tomato plants: (**A**) fruit fresh weight and (**B**) number of fruit/plant. Bars with different lower-case letters are significantly different at *p ≤* 0.05 (Duncan’s multiple range test). Vertical bars indicate standard error (SE) values.

The fruit was examined for fresh weight, size, quantity of fruits per plant, and fruit yield when collected 115 days after transplantation (Figure 6 and Figure 7). With more silver nanoparticle treatment than the control, the quantity of fruits produced per plant rose. The number of fruits per plant increased with the increase of the treatment of silver nanoparticles compared to the control. The production was increased from 20 to 36 and 21 to 37 in the case of the nadar and naqeeb plants, respectively. The plants treated with 10 ppm silver nanoparticles produced the highest number of fruit in the case of both varieties. The higher concentrations of silver nanoparticles caused the reduction of the fruit number compared to the control. More than 85% of the obtained fruit was marketable, with an average weight of 38 g. The weight of the fruit was significantly affected by applying silver nanoparticles. A maximum weight of 61.33 g with 10 ppm AgNPs in the fruit of the nadar variety and 57.67 g with 5 ppm AgNPs in the naqeeb variety was observed. The fruit harvested from plants exposed to doses of 25 and 50 ppm were not considered marketable since their average weight (18 to 20 g) was less than 30 g, and their size was 35% less than that of the control, as shown in Figure 7a. The maximum fruit yield (kg/plant) was obtained from plants treated with 10 ppm silver nanoparticles by 35–43% more than control plants. The lower concentrations of silver nanoparticles (5 and 10 ppm) significantly affected the overall growth and fruit yield of both varieties of tomato plants. In comparison, the higher concentrations caused stress and resulted in the production of unmarketable fruit.

## 4. Discussion

Silver nanoparticles (AgNPs) are often characterized by their unique physical, chemical, and biological properties [66]. These characteristics have to do with particle size, shape, and surface chemistry, all of which may be altered using synthesis techniques [67]. Using plant extracts as reducing and stabilizing agents during the nanoparticle production process is known as “green synthesis”. This method is eco-friendly, is cost-effective, and can produce nanoparticles with unique properties [68]. Many studies have been conducted on silver nanoparticles (AgNPs), which are known to exhibit a broad spectrum of antifungal actions [69]. AgNPs’ antifungal efficacy is influenced by several variables, including particle size, shape, surface charge, and concentration [41]. Due to their increased surface area-to-volume ratio, smaller AgNPs tend to have better antifungal activity than bigger particles, which improves their capacity to enter fungal cells and interact with cellular components [70]. According to Hernandez-Daz et al. [71] and Mansoor et al. [72], AgNPs interact with *Alternaria solani*’s cell membrane and induce structural damage that results in membrane permeabilization, oxidative stress, and cell death.

In the current study, the green synthesized silver nanoparticles of 22–30 nm size were proven to be an effective antifungal agent in the laboratory and under greenhouse conditions against *Alternaria solani* causing early blight disease. Even at lower concentrations, the AgNPs inhibited the development of the fungus by more than 50% compared to the control and reduced the symptoms. The higher concentrations of silver nanoparticles completely prevented the fungus growth. AgNPs applied as a foliar spray reduce the fungal growth, mycelial development, disease severity, and oxidative damage caused by *A. solani*, leading to improved plant growth and development [73]. Silver nanoparticles have been found to increase the accumulation of certain phytochemicals, such as phenols and flavonoids, which have been shown to have antifungal properties [74]. By controlling the synthesis of stress-related hormones, enhancing the plant’s antioxidant defense system, and influencing the expression of stress-responsive genes against pathogenic stress, AgNPs increase plants’ ability to withstand stress [75,76]. AgNPs lessen oxidative stress-related harm by having a high surface area to volume ratio, being highly reactive, and being able to interact with cellular components [77].

Silver nanoparticles enhance the activity of stress-tolerant enzymes, such as peroxidases (PO) and polyphenol peroxidase (PPO), and phenylalanine ammonia-lyase (PAL), which can help reduce oxidative damage and improve the plant’s stress tolerance [78,79,80]. PO is an enzyme that catalyzes the oxidation of various substrates using hydrogen peroxide as an oxidizing agent. In plants, it plays a role in the defense against biotic stress, such as pathogen attack, by generating ROS to help inactivate the pathogens [81]. Another enzyme, PPO, is involved in lignifying cell walls to reinforce plant tissues against stress [82]. Secondary metabolites including flavonoids and phenylpropanoids are biosynthesized by PAL [83]. The formation of secondary metabolites, which can function as antioxidants and can aid in the reduction of ROS, was increased as a result of AgNPs activating the PAL activity [84]. Another study found that applying AgNPs to tomato plants increased the activity of the enzymes PO, PPO, and PAL, which are crucial for the manufacturing of phytochemicals and the body’s response to pathogenic assaults [68].

The use of silver nanoparticles enhances the activity of the enzymes superoxide dismutase (SOD), catalase (CAT), and ascorbate peroxidase (APX) in plants, boosting the plant’s capacity to resist oxidative stress and maintain cellular homeostasis under stressful circumstances [85]. SOD protects the plant from oxidative stress by converting superoxide radicals into hydrogen peroxide and molecular oxygen [86]. CAT acts to decompose hydrogen peroxide into water and oxygen [87]. In comparison, APX is involved in the ascorbate-glutathione cycle and helps neutralize the plant’s hydrogen peroxide and other reactive oxygen species [88]. These enzymes are crucial in the plant’s defense mechanism against pathogen infections. By maintaining the balance of reactive oxygen species and reducing oxidative damage, SOD, CAT, and APX help the plant to overcome stress conditions and improve its overall health and productivity. It has been found that applying AgNPs to tomato plants resulted in upregulating SOD, CAT, and APX activities, reducing oxidative damage levels, and improving plant growth under early blight stress conditions [89]. AgNPs can improve the tomato crop’s growth, yield, and quality by enhancing the plant’s overall health and protecting it from disease.

The green synthesis of silver nanoparticles has positively affected tomato plant growth, development, productivity, and increased resistance to pathogens when applied as a foliar spray. In several plant species, including tomato, the use of green produced AgNPs has been demonstrated to improve the shoot length, root length, number of leaves, and leaf area [90,91]. Shoot length, root length, and plant height all increase as a result of the AgNP stimulation of cell division and elongation [92]. By promoting root growth and boosting nutrient uptake from the soil, AgNPs increase plant growth [93]. Improvements in soil nutrient uptake, greater chlorophyll content in the leaves, and better plant resiliency to external stressors are assumed to be the causes of a rise in growth characteristics [94].

Applying AgNPs increases leaves’ chlorophyll content, leading to improved photosynthetic efficiency [95,96]. AgNPs enhance photosynthetic activity by lowering oxidative stress [97] and enhancing the uptake of vital nutrients such as nitrogen, phosphorus, and potassium. They also assist in maintaining the structural integrity of chloroplasts and other cellular components involved in photosynthesis [98]. The synthesis of several secondary metabolites, including sugar, protein, alkaloids, flavonoids, and phenolic compounds, is improved when resources are more readily available [99]. It is indicated that AgNPs influence the levels of plant hormones, such as auxins and gibberellins, which regulate alkaloid biosynthesis [100]. AgNPs have been shown to modulate the activity of certain enzymes involved in the biosynthesis of alkaloids, leading to changes in alkaloid production [101]. It has been suggested that silver nanoparticles can trigger the plant’s stress response, leading to increased flavonoid synthesis [102]. These compounds have anti-inflammatory, antioxidant, and antimicrobial properties and play important roles in plant defense against pathogens and environmental stress, and they also have potential health benefits for humans [5,100].

The use of AgNPs has been reported to increase tomato output [73,103,104]. The growth and fruit output of tomato plants were shown to be enhanced by AgNPs at a dosage of 5 ppm. The study concluded that greater plant vigor and stronger antioxidant defense systems were responsible for the increased output [105]. Fruit output in tomato plants has been demonstrated to be impacted by the application of silver nanoparticles (AgNPs). Depending on the dose and length of exposure, AgNPs can have either favorable or detrimental impacts on the fruit output of tomato plants. According to certain research, utilizing AgNPs can boost plant growth and physiological functions including photosynthesis, nutrient absorption, and stress tolerance, which in turn can enhance tomato plants’ fruit output [106]. By causing oxidative stress, lowering photosynthesis, and disrupting the plant’s hormonal balance, larger concentrations of AgNPs, such as 100 or more, might, on the other hand, negatively affect tomato plant development and fruit output.

## 5. Conclusions

The present study provides compelling evidence for the potential of green synthesized AgNPs as a novel approach for enhancing the growth and yield of tomato plants and protecting them against early blight disease. The AgNPs synthesized using leaf extract of the neem tree were found to be effective in promoting plant growth, as evidenced by the significant increase in plant height, fresh and dry weight, chlorophyll, alkaloids, total soluble sugar, flavonoids, and total soluble protein of the tomato plants. Moreover, the plants treated with AgNPs exhibited a significant reduction in disease severity and disease incidence compared to the control plants, increasing the enzymatic activities of antioxidants PO, PPO, PAL, SOD, CAT, and APX, indicating their potential as an eco-friendly and sustainable solution for plant protection. These findings highlight the potential of using green synthesized AgNPs as a safe, effective, and environment friendly alternative to conventional fungicides for crop protection and yield enhancement.

## Figures and Tables

**Table 1:** The effect of green synthesized silver nanoparticles on the growth parameters of tomato plants of two varieties at vegetative and reproductive stages.

Days after Transplantation (DAT)	Treatments	Shoot Length (cm)		Root Length (cm)		No. of Leaves		Leaf Area (cm^2^)	
		Nadar	Naqeeb	Nadar	Naqeeb	Nadar	Naqeeb	Nadar	Naqeeb
55 DAT	Control	31.58 ^d^ ± 1.816	30.96 ^c^ ± 1.996	18.67 ^b^ ± 0.749	16.83 ^d^ ± 1.072	46.67 ^d,^ ± 0.863	46.67 ^d^ ± 1.176	12.92 ^c^ ± 1.934	14.82 ^b, c^ ± 1.008
	F	34.37 ^c,d^ ± 1.050	31.25 ^c^ ± 0.517	23.38 ^b^ ± 1.888	17.60 ^c, d^ ± 0.734	51.67 ^c^ ± 1.176	47.67 ^d^ ± 1.481	13.37 ^c^ ± 1.655	15.63 ^b^ ± 1.872
	5	51.50 ^a,b^ ± 2.178	44.94 ^b^ ± 1.137	29.03 ^a^ ± 1.604	26.17 ^a, b^ ± 1.187	75.33 ^a^ ± 1.726	65.33 ^c^ ± 1.439	20.55 ^a^ ± 0.848	22.30 ^a^ ± 1.078
	10	52.81 ^a^ ± 1.899	53.85 ^a^ ± 0.831	29.30 ^a^ ± 2.063	27.52 ^a^ ± 2.402	78.00 ^a^ ± 1.565	80.67 ^a^ ± 1.422	20.97 ^a^ ± 0.969	25.02 ^a^ ± 2.073
	15	45.95 ^b^ ± 0.523	49.67 ^a,b^ ± 1.360	22.67 ^b^ ± 1.732	25.28 ^a, b^ ± 2.199	63.33 ^b^ ± 0.863	73.00 ^b^ ± 2.463	16.43 ^b^ ± 2.198	22.60 ^a^ ± 1.452
	20	38.20 ^c^ ± 0.877	45.50 ^b^ ± 1.398	21.66 ^b^ ± 0.801	22.25 ^b, c^ ± 2.048	53.67 ^c^ ± 2.670	62.67 ^c^ ± 1.422	12.50 ^c^ ± 1.545	14.39 ^b,c^ ± 0.772
	25	30.51 ^d, e^ ± 2.857	33.49 ^c^ ± 3.001	19.66 ^b^ ± 1.157	19.00 ^c, d^ ± 1.090	45.67 ^d^ ± 1.305	49.33 ^d^ ± 1.176	10.45 ^cd^ ± 2.292	13.29 ^b,c^ ± 1.789
	50	25.74 ^e^ ± 2.107	28.54 ^c^ ± 3.160	19.03 ^b^ ± 0.927	17.03 ^c, d^ ± 0.995	40.67 ^e^ ± 1.422	47.33 ^d^ ± 1.768	8.30 ^d^ ± 1.539	10.97 ^c^ ± 0.662
85 DAT	Control	38.37 ^e^ ± 2.794	44.80 ^d^ ± 2.268	26.57 ^d^ ± 1.583	27.77 ^d^ ± 1.669	65.33 ^d^ ± 2.336	64.67 ^d^ ± 2.336	27.87 ^d, e^ ± 1.293	29.67 ^d^ ± 0.915
	F	38.17 ^e^ ± 0.919	47.37 ^d^ ± 2.489	27.20 ^d^ ± 1.740	28.77 ^d^ ± 1.090	65.67 ^d^ ± 1.766	67.67 ^d^ ± 2.732	28.13 ^d, e^ ± 2.605	30.87 ^c,d^ ± 1.685
	5	68.43 ^a^ ± 0.998	76.87 ^a^ ± 2.805	44.17 ^a^ ± 1.848	49.10 ^a^ ± 2.282	105.00 ^a^ ± 2.084	111.00 ^a^ ± 2.046	42.87 ^a^ ± 1.358	46.10 ^a^ ± 0.851
	10	63.80 ^b^ ± 1.695	72.00 ^a, b^ ± 0.980	45.27 ^a^ ± 2.285	43.57 ^b^ ± 2.045	103.67 ^a^ ± 2.188	103.00 ^b^ ± 1.529	39.57 ^a, b^ ± 1.435	44.00 ^a, b^ ± 0.834
	15	56.47 ^c^ ± 1.726	66.90 ^b, c^ ± 1.336	40.57 ^a,b^ ± 2.285	36.77 ^c^ ± 0.865	94.33 ^b^ ± 1.766	97.33 ^b^ ± 2.966	35.33 ^b, c^ ± 1.076	42.83 ^b^ ± 2.732
	20	48.13 ^d^ ± 0.932	63.23 ^c^ ± 1.783	36.20 ^c^ ± 0.746	35.17 ^c^ ± 1.899	72.67 ^c^ ± 1.203	88.67 ^c^ ± 1.766	32.47 ^c, d^ ± 3.386	32.67 ^c^ ± 1.074
	25	40.97 ^e^ ± 1.005	50.97 ^d^ ± 0.711	33.33 ^d^ ± 0.261	29.00 ^c, d^ ± 1.395	68.33 ^c, d^ ± 1.766	67.33 ^d^ ± 0.883	25.77 ^e^ ± 2.555	28.43 ^d^ ± 1.665
	50	33.33 ^f^ ± 1.220	45.33 ^d^ ± 2.514	27.47 ^d^ ± 2.123	28.13 ^d^ ± 1.339	57.67 ^e^ ± 1.455	65.67 ^d^ ± 1.455	24.60 ^e^ ± 1.644	25.77 ^e^ ± 2.167

The treatment mean for each treatment is the average of the three replicates; mean values with different lower-case letters are significantly different at *p ≤* 0.05 (Duncan’s multiple range test). ± values represent standard error (SE).

**Table 2:** The effect of green synthesized silver nanoparticles on the biomass production of tomato plants of two varieties at vegetative and reproductive stages.

Days after Transplantation (DAT)	Treatments	Shoot Fresh Weight (g)		Root Fresh Weight (g)		Shoot Dry Weight (g)		Root Dry Weight (g)	
		Nadar	Naqeeb	Nadar	Naqeeb	Nadar	Naqeeb	Nadar	Naqeeb
55 DAT	Control	28.71 ^c,d^ ± 1.680	28.75 ^c^ ± 1.626	9.09 ^d^ ± 0.393	8.56 ^c^ ± 0.598	8.36 ^c,d^ ± 1.217	6.79 ^b^ ± 0.582	3.33 ^c^ ± 0.172	3.36 ^d^ ± 0.412
	F	29.88 ^c,d^ ± 1.733	29.51 ^c^ ± 0.808	11.23 ^b,c^ ± 0.338	8.89 ^c^ ± 0.524	8.91 ^c,d^ ± 0.362	7.01 ^b^ ± 0.525	4.04 ^a,b,c^ ± 0.260	3.43 ^d^ ± 0.137
	5	48.17 ^a^ ± 1.605	42.30 ^b^ ± 1.581	14.06 ^a^ ± 1.369	13.34 ^a,b^ ± 0.550	13.43 ^a,b^ ± 1.605	10.22 ^a^ ± 0.695	5.00 ^a,b^ ± 0.169	4.79 ^a,b^ ± 0.474
	10	49.89 ^a^ ± 1.775	51.11 ^a^ ± 0.526	14.26 ^a^ ± 0.901	13.85 ^a^ ± 1.345	15.00 ^a^ ± 1.775	11.90 ^a^ ± 0.897	5.25 ^a^ ± 0.066	5.42 ^a^ ± 0.422
	15	41.47 ^b^ ± 0.492	48.08 ^a^ ± 2.679	11.35 ^b^ ± 0.831	12.82 ^a,b^ ± 0.976	11.25 ^b,c^ ± 0.395	10.70 ^a^ ± 1.228	4.29 ^a,b,c^ ± 0.639	4.86 ^b,c^ ± 0.356
	20	33.38 ^c^ ± 0.817	41.92 ^b^ ± 1.348	10.80 ^b,c,d^ ± 0.288	10.90 ^b,c^ ± 1.388	9.29 ^c,d^ ± 0.817	10.19 ^a^ ± 0.348	3.81 ^a,b,c^ ± 0.192	4.22 ^c^ ± 0.145
	25	28.98 ^c,d^ ± 2.313	29.74 ^c^ ± 1.416	9.95 ^b,c,d^ ± 0.252	9.73 ^c^ ± 0.609	8.76 ^c,d^ ± 0.407	7.41 ^b^ ± 0.366	3.48 ^b,c^ ± 0.128	3.75 ^c,d^ ± 0.601
	50	24.62 ^d^ ± 2.039	27.32 ^c^ ± 2.146	9.35 ^c,d^ ± 0.374	8.89 ^c^ ± 0.348	7.18 ^d^ ± 1.251	6.36 ^b^ ± 0.148	3.50 ^b,c^ ± 0.450	3.47 ^d^ ± 0.253
85 DAT	Control	39.63 ^e^ ± 0.613	45.04 ^d^ ± 2.117	13.20 ^d^ ± 0.853	12.69 ^e^ ± 0.635	8.24 ^d^ ± 0.183	7.90 ^b^ ± 0.432	3.33 ^c^ ± 0.234	3.09 ^c^ ± 0.168
	F	40.30 ^e^ ± 0.966	47.70 ^d^ ± 2.624	13.57 ^c,d^ ± 1.217	13.46 ^d,e^ ± 0.422	8.62 ^d^ ± 0.461	8.67 ^b^ ± 0.291	3.36 ^c^ ± 0.277	3.23 ^c^ ± 0.198
	5	70.07 ^a^ ± 0.866	79.14 ^a^ ± 5.411	23.59 ^a^ ± 0.693	21.81 ^a^ ± 1.095	13.93 ^a^ ± 0.433	13.58 ^a^ ± 0.575	5.47 ^a^ ± 0.167	5.34 ^a^ ± 0.344
	10	65.07 ^b^ ± 1.998	74.44 ^a,b^ ± 1.190	21.77 ^a^ ± 1.204	19.73 ^b^ ± 1.066	13.50 ^a,b^ ± 0.495	13.25 ^a^ ± 0.575	5.59 ^a^ ± 0.404	4.67 ^b^ ± 0.332
	15	57.97 ^c^ ± 2.245	69.73 ^b,c^ ± 1.940	17.08 ^b^ ± 0.798	15.69 ^c^ ± 0.197	12.60 ^b^ ± 0.477	13.64 ^a^ ± 1.084	4.20 ^b^ ± 0.261	3.77 ^c^ ± 0.085
	20	49.10 ^d^ ± 1.125	63.82 ^c^ ± 2.324	16.18 ^b,c^ ± 0.758	15.03 ^c,d^ ± 0.568	10.81 ^c^ ± 0.395	11.81 ^a^ ± 0.474	3.97 ^b,c^ ± 0.114	3.60 ^c^ ± 0.239
	25	41.87 ^e^ ± 1.505	51.14 ^d^ ± 1.154	14.69 ^b,c,d^ ± 0.474	13.25 ^d,e^ ± 0.508	8.86 ^d^ ± 0.404	9.38 ^b^ ± 0.962	3.77 ^b,c^ ± 0.113	3.19 ^c^ ± 0.118
	50	34.77 ^f^ ± 2.294	45.27 ^d^ ± 2.358	13.71 ^c,d^ ± 0.860	13.03 ^d,e^ ± 0.373	7.82 ^d^ ± 0.163	8.03 ^b^ ± 0.253	3.36 ^c^ ± 0.151	3.10 ^c^ ± 0.128

The treatment mean for each treatment is the average of the three replicates; mean values with different lower-case letters are significantly different at *p ≤* 0.05 (Duncan’s multiple range test). ± values represent standard error (SE).

**Table 3:** The effect of green synthesized silver nanoparticles on photosynthetic pigments of tomato plants of two varieties at vegetative and reproductive stages.

Days after Transplantation (DAT)	Treatments	Chl a (mg/g FW)		Chl b (mg/g FW)		Carotenoid (mg/g FW)	
		Nadar	Naqeeb	Nadar	Naqeeb	Nadar	Naqeeb
55 DAT	Control	1.415 ^c^ ± 0.063	1.958 ^b^ ± 0.076	0.322 ^c^ ± 0.019	0.419 ^b^ ± 0.017	0.424 ^c^ ± 0.038	0.837 ^b^ ± 0.018
	F1	1.465 ^c^ ± 0.099	2.098 ^b^ ± 0.058	0.337 ^c^ ± 0.015	0.448 ^b^ ± 0.028	0.435 ^c^ ± 0.036	0.890 ^b^ ± 0.041
	5	2.263 ^a^ ± 0.040	3.005 ^a^ ± 0.058	0.510 ^a^ ± 0.035	0.625 ^a^ ± 0.051	0.660 ^a^ ± 0.039	1.247 ^a^ ± 0.062
	10	2.285 ^a^ ± 0.098	3.250 ^a^ ± 0.065	0.520 ^a^ ± 0.028	0.707 ^a^ ± 0.018	0.680 ^a^ ± 0.028	1.377 ^a^ ± 0.047
	15	1.881 ^b^ ± 0.077	2.962 ^a^ ± 0.050	0.420 ^b^ ± 0.013	0.635 ^a^ ± 0.017	0.563 ^b^ ± 0.039	1.276 ^a^ ± 0.064
	20	1.365 ^c^ ± 0.059	1.994 ^b^ ± 0.064	0.320 ^c^ ± 0.018	0.426 ^b^ ± 0.025	0.423 ^c^ ± 0.049	0.864 ^b^ ± 0.027
	25	1.112 ^d^ ± 0.038	1.746 ^b,c^ ± 0.063	0.260 ^d^ ± 0.017	0.373 ^b,c^ ± 0.014	0.335 ^d^ ± 0.038	0.795 ^b^ ± 0.037
	50	1.038 ^d^ ± 0.033	1.556 ^c^ ± 0.089	0.227 ^d^ ± 0.016	0.324 ^c^ ± 0.015	0.311 ^d^ ± 0.066	0.702 ^c^ ± 0.032
85 DAT	Control	2.034 ^b,c^ ± 0.048	2.119 ^c,d^ ± 0.045	0.774 ^d,e,f^ ± 0.027	0.762 ^b,c^ ± 0.027	0.699 ^e^ ± 0.042	0.953 ^b^ ± 0.023
	F1	2.086 ^b,c^ ± 0.062	2.212 ^c^ ± 0.067	0.787 ^d,e^ ± 0.026	0.799 ^b,c^ ± 0.028	0.707 ^e^ ± 0.039	1.008 ^b^ ± 0.051
	5	3.135 ^a^ ± 0.076	3.351 ^a^ ± 0.056	1.193 ^a^ ± 0.061	1.198 ^a^ ± 0.044	1.069 ^a^ ± 0.063	1.485 ^a^ ± 0.045
	10	2.927 ^a^ ± 0.083	3.099 ^a,b^ ± 0.081	1.091 ^a,b^ ± 0.037	1.134 ^a^ ± 0.045	0.977 ^b^ ± 0.019	1.420 ^a^ ± 0.049
	15	2.336 ^b^ ± 0.034	3.006 ^b^ ± 0.028	0.984 ^b,c^ ± 0.009	1.113 ^a^ ± 0.061	0.892 ^c^ ± 0.033	1.359 ^a^ ± 0.089
	20	2.114 ^b,c^ ± 0.079	2.301 ^c^ ± 0.096	0.890 ^c,d^ ± 0.018	0.868 ^b^ ± 0.037	0.808 ^d^ ± 0.030	1.070 ^b^ ± 0.032
	25	1.800 ^c^ ± 0.043	2.053 ^c,d^ ± 0.054	0.731 ^e,f^ ± 0.095	0.714 ^c^ ± 0.019	0.646 ^e,f^ ± 0.012	0.916 ^b^ ± 0.032
	50	1.769 ^c^ ± 0.086	1.870 ^d^ ± 0.038	0.663 ^f^ ± 0.018	0.665 ^c^ ± 0.013	0.615 ^f^ ± 0.022	0.829 ^c^ ± 0.022

The treatment mean for each treatment is the average of the three replicates; mean values with different lower-case letters are significantly different at *p ≤* 0.05 (Duncan’s multiple range test). ± values represent standard error (SE).

## Data Availability

The paper contains the original contributions that were presented in the study. The appropriate author can be contacted for more information.
